# Stimulation of the Production of Prostaglandin E_2_ by Ethyl Gallate, a Natural Phenolic Compound Richly Contained in Longan

**DOI:** 10.3390/biom8030091

**Published:** 2018-09-06

**Authors:** Hui Rong Wang, Hao Chen Sui, Yan Yan Ding, Bao Ting Zhu

**Affiliations:** 1 Department of Biology, Southern University of Science and Technology, Shenzhen 518055, China; 11553012@mail.sustc.edu.cn (H.R.W.); dingyy@sustc.edu.cn (Y.Y.D.); 2 School of Science and Engineering, The Chinese University of Hong Kong, Shenzhen 518172, China; 115010223@link.cuhk.edu.cn

**Keywords:** ethyl gallate, cyclooxygenase, peroxidase activity, mechanism of catalytic activation

## Abstract

Ethyl gallate is a phenolic compound richly contained in Longan. In traditional Chinese medicine, Longan is widely known as a fruit with “hot” properties, with a tendency to promote inflammatory and certain other responses. The mechanism for its proinflammatory as well as health beneficial effects is poorly understood. Based on our earlier observation that certain natural phenolic compounds can serve as reducing cosubstrates for cyclooxygenases (COXs), we sought to test a hypothesis that ethyl gallate may activate the catalytic activity of the COX enzymes. Results from studies using cultured cells and animals show that ethyl gallate can activate the production of prostaglandin E_2_, a representative prostaglandin tested in this study. Computational analysis indicates that ethyl gallate can activate the peroxidase active sites of COX-1 and COX-2 by serving as a reducing cosubstrate. The effect of ethyl gallate is abrogated by galangin, which is known to bind to the same peroxidase active sites of COX-1 and COX-2 as a competitive inhibitor. The findings of this study offer support for a novel hypothesis that the proinflammatory as well as health beneficial effects of Longan may be partly attributable to the activation of COX-1 and COX-2 by ethyl gallate.

## 1. Introduction

Cyclooxygenase 1 and 2 (COX-1 and COX-2) are key enzymes involved in arachidonic acid (AA) metabolism, resulting in the formation of important bio-mediators, including prostaglandins (PGs), prostacyclins, thromboxanes, and others [1,2,3]. Because these bio-mediators affect many pathological and/or physiological processes, pharmacological modulation of the catalytic activity of the COX enzymes has become an effective strategy in treating certain medical conditions [2,4,5,6,7].

Longan (also known as *Dimocarpus longan*) is a member of the soapberry family (Sapindaceae). It is grown extensively in China and South East Asia, as well as in Australia, Florida (United States of America (U.S.A.)), southern Europe, and southern Africa [8,9]. In traditional Chinese medicine, Longan is widely known as a fruit with “hot” properties (i.e., it has a tendency to promote inflammatory and certain other responses) [10,11,12,13]. Studies have shown that Longan contains high levels of ethyl gallate [14,15] and other phenolic compounds [15,16,17]. Ethyl gallate is also found in other plants, such as walnuts [18] and *Terminalia myriocarpa* [19], and it is also present in wine [20].

The mechanism underlying the proinflammatory and health beneficial effects of Longan is poorly understood at present. In earlier studies [21,22], we showed, for the first time, that some natural phenolics, such as quercetin and myricetin, can serve as reducing cosubstrates of COX-1 and COX-2 and activate their catalytic activity. This phenomenon was confirmed in an in vitro cell culture model [21] and an in vivo animal model [23]. Notably, these compounds are effective in activating the COX enzymes in cultured cells with effective concentrations in the nM range [21]. 

In the present study, we sought to determine whether ethyl gallate can modulate PG biosynthesis in cultured cells and intact animals. The possible mechanism for its modulating effect was explored using computational modeling approaches that probe their binding interaction with the COX-1 and COX-2 enzymes.

## 2. Materials and Methods

### 2.1. Chemicals and Reagents

Ethyl gallate (purity > 99%), arachidonic acid (AA), galangin, lipopolysaccharide (LPS; from *Escherichia coli*, serotype 055:b5), and Dulbecco’s modified Eagle’s medium (DMEM) were obtained from Sigma-Aldrich (St. Louis, MO, USA). Fetal bovine serum (FBS) was purchased from GIBCO-Thermo Fisher Scientific (Waltham, MA, USA). The anti-COX-1 antibody and anti-COX-2 antibody were obtained from Abcam (Cambridge, UK), and the anti-glyceraldehyde 3-phosphate dehydrogenase (GAPDH) antibody was obtained from Cell Signaling Technology (Danvers, MA, USA). The enzymatic immunoassay (EIA) kit for measurement of prostaglandin E_2_ (PGE_2_) was obtained from Cayman Chemical (Ann Arbor, MI, USA).

### 2.2. In Vitro Cell Culture Experiments

The RAW264.7 cell line (murine macrophages) was purchased from Shanghai Institute of Biochemistry and Cell Biology, Chinese Academy of Sciences (CAS), and maintained in DMEM containing l-glutamine, glucose, and sodium bicarbonate supplemented with 10% fetal bovine serum at 37 °C under 5% CO_2_. As described in our earlier studies [21,24], the cells were first treated with LPS (at 1 µg/mL) for 2 h to increase the expression levels of the COX enzymes (almost exclusively COX-2). Then, the culture medium was removed and replaced with 300 µL of serum-free medium in the absence or presence of different concentrations of ethyl gallate. After culturing for additional 2 h, the medium was collected for measurement of PGE_2_ using an EIA kit (Cayman Chemical) according to the detailed procedures provided by the manufacturers.

### 2.3. In Vivo Animal Experiments

All procedures involving the use of live animals as described in the present study were approved by the Institutional Animal Care and Use Committee (approval number: SUSTC-G-2014009), and the guidelines for humane treatment of animals accepted by the National Institutes of Health (U.S.A.) were followed. The male Sprague-Dawley rats (four to five-week-old, specific pathogen-free) were obtained from Guangdong Medical Laboratory Animal Center (Guangdong, China) and were maintained in our institute’s central animal facility. The animals were fed a standard rodent chow purchased from Beijing Keaoxieli (Beijing, China). After arrival, the animals were allowed to acclimatize for one week prior to being used in the experimentation. The animals were housed under constant conditions of temperature (20 ± 1 °C) and 12-h light/12-h dark cycle and had free access to food and water.

The male rats were divided into the following groups (five animals per group): the control group (treated with the vehicle only) and the ethyl gallate treatment group (receiving ethyl gallate at 6 mg/kg body weight, dissolved in 1.5 mL of 1% methyl cellulose). Ethyl gallate was given via gastric intubation, and the control animals were given 1.5 mL of vehicle only. The use of the 6 mg/kg body weight dose of ethyl gallate was based on an initial dose-escalating experiment, which showed that this in vivo oral dose causes a consistent and reproducible increase in PGE_2_ plasma levels in rats. Whole blood samples were collected through tail bleeding at different timepoints following ethyl gallate or vehicle administration and stored in small vials containing heparin. Plasma was prepared from the collected blood by centrifugation. The plasma level of PGE_2_ was determined using an EIA kit (Cayman Chemical) according to the manufacturer’s instructions.

### 2.4. Molecular Docking Analysis of the Binding of Ethyl Gallate with COX Enzymes

In this study, a Dell PowerEdge R730 Server with the *Discovery Studio* modeling software (Version 2007; Accelrys, San Diego, CA, USA) was used for various computational analyses, as described below.

#### 2.4.1. Protein Processing

We used the X-ray structures of sheep COX-1 (PDB code: 1q4g [25]) and mouse COX-2 (PDB code: 3nt1 [4]) as templates for docking analysis. It is of note that in these two COX structures, protoporphorin IX with Fe^IV^ inside (P^+^Fe^IV^) is already present as an integral component, and the ion atom is set as Fe^4+^. As part of the protein preparation step, all small molecules except P^+^Fe^IV^ that are non-covalently attached to the COX proteins were removed, and then, the amino acid residues in the protein structure were re-numbered according to the correct known sequences. The *Clean Protein* module in *Discovery Studio* was used to complete the side chains for the amino acid residues, correct bonding and bond orders, and add hydrogens back. Lastly, the *Prepare Protein* module in *Discovery Studio* was used for protein preparation under the *CHARMm* force field setting.

#### 2.4.2. Ligand Processing

**Figure 1:**
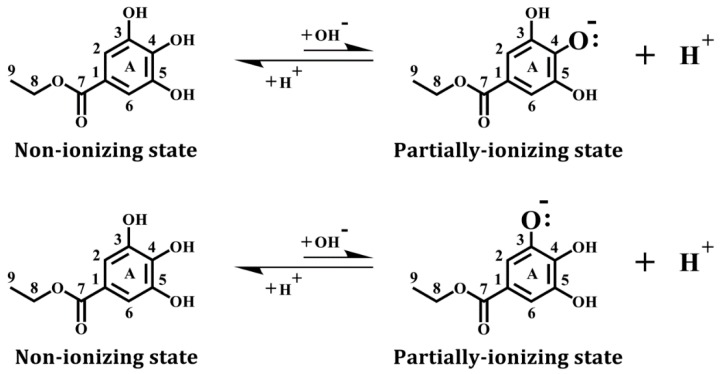
Chemical structure of ethyl gallate, a natural phenolic compound. For instance, after ionization (deprotonation) of the C-4-OH hydroxyl group, its oxygen atom in this hydroxyl group carries a negative charge with an additional electron retained. Similar ionization can also occur with the C-3-OH group, as well as the C-5-OH group.

The structure of ethyl gallate was downloaded from the Protein Data Bank and minimized with the *CHARMm* force field. In addition, we used the *Prepare Ligands* module to generate ethyl gallate in a non-ionizing state and two partially-ionizing states. The non-ionizing state has all hydrogens in the three phenolic hydroxyl groups retained, whereas the ionizing states each have one proton removed (i.e., deprotonation) from one of the three hydroxyl groups in ethyl gallate, which include C-3-OH (equivalent to C-5-OH) and C-4-OH (see Figure 1).

#### 2.4.3. Flexible Docking

For flexible docking, we used the *Find Sites* from *Receptor Cavities* module to identify the binding site in the prepared 1q4g COX-1 and 3nt1 COX-2 structures. According to our earlier study, the target site is the peroxidase active site in these two COX proteins [22]. We selected all amino acid residues within a 5 Å reach of the target site and allowed them to have flexible side chains. The *SBD Site Sphere* is centered at the target site and then expanded to 13-radius size. Under the *Flexible Docking* mode, with the conformation method set to *BEST*, the *Simulated Annealing* docking method was then applied to dock ethyl gallate into the target sites of COX-1 and COX-2. Notably, two flexible docking modes were separately executed for COX-1 and COX-2, corresponding to the two different ionizing states of ethyl gallate. The whole structure of each COX protein was further minimized with the *CHARMm* force field.

#### 2.4.4. Calculation of Binding Energy

Following the completion of the flexible docking procedure, the *Calculate Binding Energies* module in *Discovery Studio* was used to find the complexes with the lowest binding energy values. According to *Discovery Studio*, the free energy for the binding interaction between a protein and its ligand is estimated from the free energies of the complex, the protein, and the ligand. These free energy values were separately calculated using the *CHARMm* force field and the Poisson–Boltzmann equation with non-polar surface area (PBSA) method [26]. In this approach, the Poisson–Boltzmann equation is solved numerically on a three-dimensional (3D) grip, and the calculated electrostatic potential is used to estimate the electrostatic solvation free energy. The ligand conformational entropy is also considered during the free binding energy calculation. The following equation is used to calculate the binding energy (Δ*G*_binding_) between ethyl gallate and the COX-1 or COX-2 protein:

Δ*G*_binding_ = *G*_complex_ ‒ (*G*_COX_ + *G*_ligand_)

where *G*_complex_ is the absolute free energy of the complex, *G*_COX_ is the absolute free energy of the COX protein, and *G*_ligand_ is the absolute free energy of the ligand [27,28]. The Δ*G*_binding_ value is used to reflect the relative interaction affinity between the COX enzyme and ethyl gallate. The free energy of each term is estimated as a sum of the following five terms:

*G* = <*G*_intra_> + <*G*_inter_> + <*G*_pol_> + <*G*_np_> − *T*ΔS

where <*G*_intra_> is the intramolecular energy of the molecule, <*G*_inter_> is the intermolecular energy of the molecule, <*G*_pol_> is the polar contribution to solvation free energy, <*G*_np_> is the nonpolar contribution to solvation free energy, and (‒*T*ΔS) is the entropic contribution (set at 298.15 *K*).

## 3. Results 

### 3.1. Effect of Ethyl Gallate on PGE_2_ Production In Vitro and In Vivo

#### 3.1.1. In Vitro Studies

**Figure 2:**
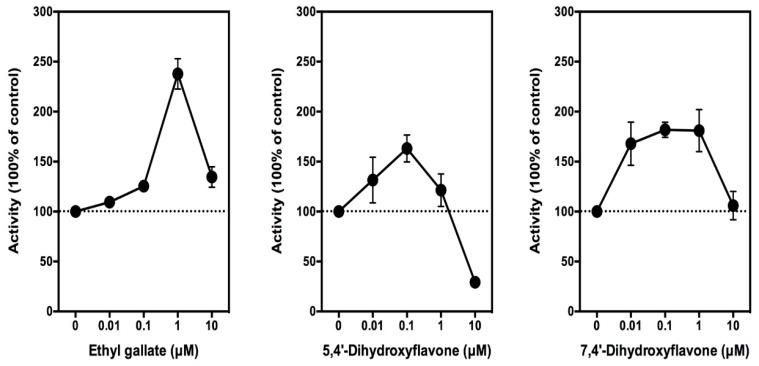
Effect of ethyl gallate, 5,4′-dihydroxyflavone, and 7,4′-dihydroxyflavone on prostaglandin E_2_ (PGE_2_) release from lipopolysaccharide (LPS)-pretreated RAW264.7 cells. Cells were pretreated with 1 μg/mL of LPS for 2 h to induce Cyclooxygenase 2 (COX-2) expression, and then, the culture media were removed and replaced with 300 μL of serum-free medium containing the test compound ethyl gallate for another 2 h. The following concentrations of ethyl gallate were used: 0.01, 0.1, 1, and 10 μM. The levels of PGE_2_ were measured using an enzymatic immunoassay (EIA) kit (Cayman Chemical). The PGE_2_ level in the control group is 1.5 ng/mL. Each point was the mean ± standard deviation (SD) of triplet determinations.

As described in our recent studies [21,24], to determine whether ethyl gallate can modulate PG production in cultured RAW264.7 cells, these cells need to be first stimulated with 1 µg/mL of LPS for 2 h to induce COX protein expression, as well as PG production. Following LPS pretreatment for 2 h, the medium is removed and replaced with 300 µL of serum-free DMEM in the absence or presence of different concentrations (0.01, 0.1 1, 10, and 100 µM) of ethyl gallate. After additional incubation for 2 h, the culture media are collected for the measurement of PGE_2_ (a representative PG determined in this study) using an EIA kit. We find that ethyl gallate at 10 nM starts to show a weak stimulatory effect on PGE_2_ production, and this stimulation reaches a plateau when ethyl gallate is present at 100–1000 nM concentrations. The maximal stimulation of PGE_2_ production by ethyl gallate is seen at 1 µM concentration, which is approximately 140% above the control level (Figure 2, left). Notably, when the concentration of ethyl gallate further increases to 10 μM, PGE_2_ production is markedly diminished. A similar phenomenon was also observed in our earlier study with several other reducing cosubstrates [21]. For comparison, we also tested 5,4’-dihydroxyflavone and 7,4’-dihydroxyflavone (two analogs of quercetin) for their effect on PGE_2_ production in this cell culture model as a positive comparison. We found that these two compounds stimulate the production of PGE_2_ in a similar manner as ethyl gallate but with a somewhat weaker efficacy (Figure 2, middle and right).

**Figure 3:**
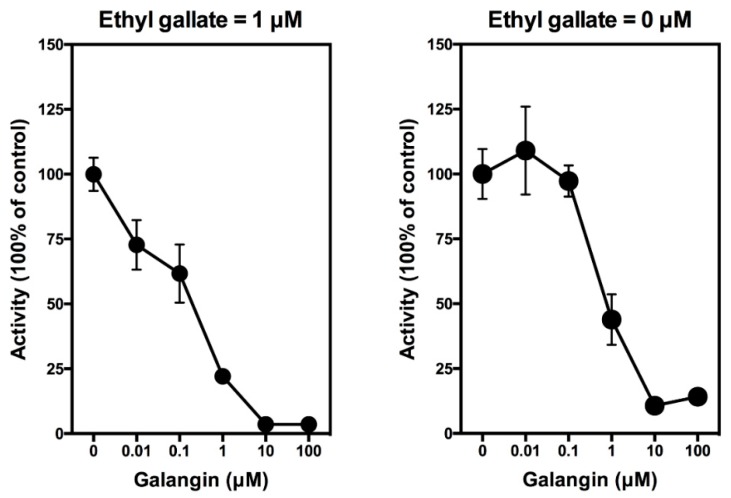
Effect of galangin on ethyl gallate-stimulated PGE_2_ release from LPS-pretreated RAW264.7 cells. Cells were pretreated with 1 μg/mL of LPS for 2 h to induce COX-2 expression, and then, the culture media were removed and replaced with 300 μL of serum-free medium containing 1 µM ethyl gallate plus different concentrations (0.01, 0.1, 1, and 10 µM) of galangin for another 2 h. The levels of PGE_2_ were measured using an EIA kit (Cayman Chemical). The PGE_2_ level in the control group is 1.5 ng/mL. Each point was the mean ± SD of triplet determinations.

In this study, we showed that galangin can abrogate the stimulation of PGE_2_ production by ethyl gallate in a concentration-dependent manner, with an *IC*_50_ value (50% inhibition concentration) of approximately 0.4 μM (Figure 3, left). When galangin was added alone to the LPS-pretreated RAW264.7 cells in the culture, it also inhibited the baseline production of PGE_2_ in a similar manner with an *IC*_50_ value of approximately 0.7 μM (Figure 3, right). It is of note that similar observations were also made in our recent study [29].

#### 3.1.2. In Vivo Studies

We also determined the effect of ethyl gallate on the plasma levels of PGE_2_ by using normal male Sprague–Dawley rats as an in vivo model. This animal model was chosen for this purpose, because we have successfully used it in similar studies to determine the effect of other natural compounds on plasma and tissue levels of several PG products [21,24]. We showed earlier that oral administration of these natural compounds to normal rats can effectively increase the tissue and/or blood levels of PG products in vivo [21,24]. 

**Figure 4:**
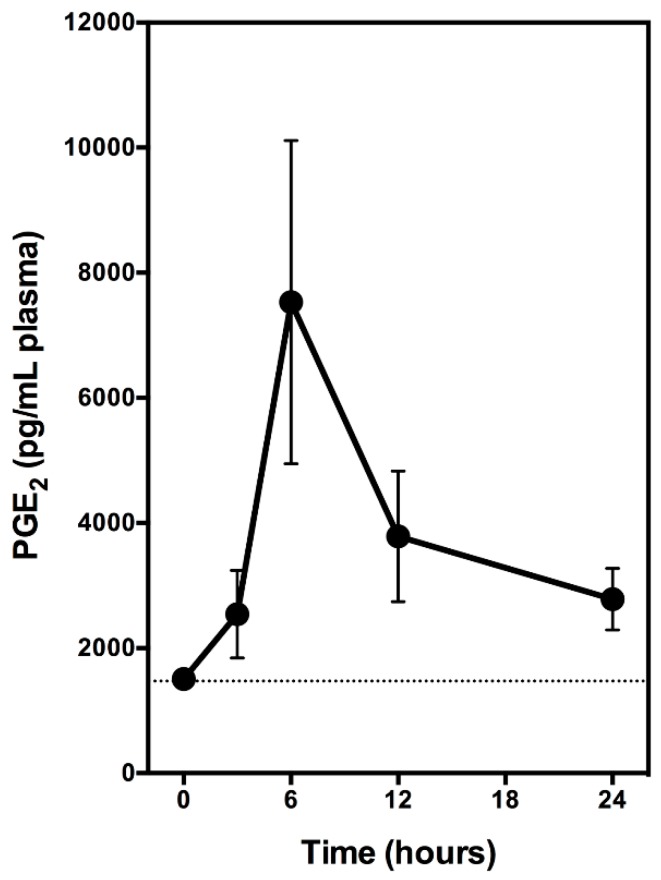
The time course for the stimulatory effect of ethyl gallate on the plasma levels of PGE_2_ in rats. Male Sprague-Dawley rats were given oral administration of ethyl gallate alone (6 mg/kg body weight), and the control animals were given vehicle treatment only. Blood samples were collected from tail bleeding at 3, 6, 9, 12, and 24 h after oral administration. The plasma was immediately prepared and stored at −80 °C. The plasma levels of PGE_2_ were measured using an EIA kit (Cayman Chemical). Data represent mean ± SD. (*N* = 5).

In this experiment, the animals received a single oral dose of ethyl gallate alone (at 6 mg/kg body weight). Blood samples were collected through tail bleeding at selected timepoints, and the plasma samples were prepared and used for PGE_2_ measurement. We find that oral administration of ethyl gallate alone markedly increases the plasma level of PGE_2_ in a time-dependent manner (Figure 4). Plasma PGE_2_ level increased significantly at 3 h after administration, and peaks at approximately 6 h after administration, with a maximal increase by approximately four-fold of the control level (Figure 4). 

In summary, in vitro experiments using cultured cells and in vivo experiments using normal male rats as an animal model both show that ethyl gallate can stimulate the production of PGE_2_. This effect is abrogated by galangin, which is an inhibitor of the COX peroxidase activity by blocking the effect of the reducing cosubstrate.

### 3.2. Computational Docking Analysis of Ethyl Gallate Binding Inside the Peroxidase Active Sites of COX-1 and COX-2

We used sheep COX-1 (PDB code: 1q4g [25]) and mouse COX-2 (PDB code: 3nt1 [4]) proteins as templates to model how ethyl gallate binds inside the peroxidase active sites of these two enzymes. Three-dimensional (3D) structural models of these two proteins were prepared by *Discovery Studio*. Using these structural models, we then docked ethyl gallate in three different ionizing states (one non-ionizing state vs two partially-ionizing states) into the peroxidase active sites of COX-1 and COX-2. The results are described below.

#### 3.2.1. COX-1

**Figure 5:**
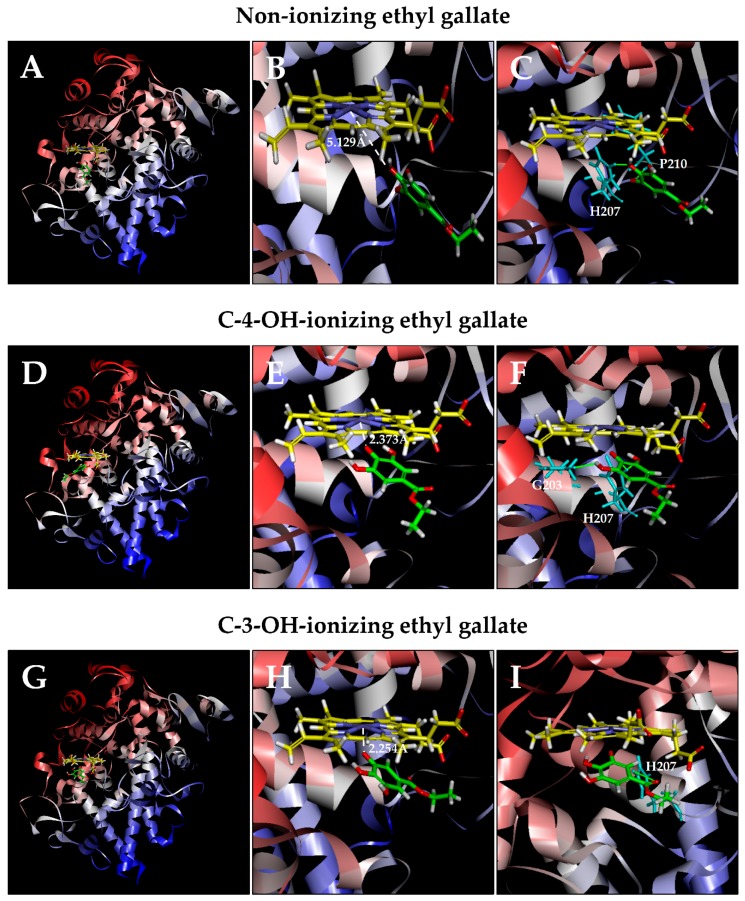
Molecular docking analysis of the binding interaction of ethyl gallate with the peroxidase active site of the COX-1 protein. Panels **A**, **D**, and **G**: The dominant docking result for non-ionizing (**A**), C-4-OH-ionizing (**D**), and C-3-OH-ionizing (**G**) ethyl gallate inside the peroxidase active site of COX-1. The protein structure is shown in a flat ribbon format. In P^+^Fe^IV^, carbon is colored in yellow, nitrogen in blue, oxygen in red, hydrogen in white, and iron in bice. In ethyl gallate, carbon is colored in green, oxygen in red, and hydrogen in white. Panels **B**, **E**, and **H**: The same structures as in panels **A**, **D**, and **G** with a white dashed line added to indicate the distance between Fe^4+^ ion and O of one of the OH groups in ethyl gallate. Panels **C**, **F**, and **I**: Suggested potential hydrogen bonds (green lines) between the non-ionizing (**C**), C-4-OH-ionizing (**F**), or C-3-OH-ionizing (**I**) ethyl gallate and the amino acid residues (Gln203, His207, Phe210) in the peroxidase site. The amino acid residues are colored in light blue.

Computational docking analysis of ethyl gallate in a non-ionizing state suggests that it can bind inside the peroxidase active site of COX-1 in two possible binding modes: one with its A-ring inside the peroxidase site facing P^+^Fe^IV^, and the other one with its side chain structure inside the peroxidase site. Based on the binding energy Δ*G*_binding_ values (Table 1), it is predicted that the binding mode with its A-ring inside is the dominant binding pose (Δ*G*_binding_ of −3.652 *k*_cal_/mol). However, in this binding pose, most hydroxyl groups of ethyl gallate are not too close to the Fe ion of P^+^Fe^IV^, and the closest one is 8.762 Å (Figure 5A,B). These results suggest that this binding pose is an inactive pose and would not be able to effectively transfer its electrons to P^+^Fe^IV^ for reduction. For poses ranked 2–10, all hydroxyl groups of ethyl gallate are slightly farther away from the Fe ion of P^+^Fe^IV^ compared with the dominant binding pose.

Potential hydrogen bonds between COX-1 and ethyl gallate in its dominant binding pose, as suggested by the *Receptor–Ligand Hydrogen Bonds* module, are shown in Figure 5C, which involve two amino acid residues: one with Phe210 (1.883 Å) and one with His207 (1.335 Å).

It is predicted that under physiological conditions, a small fraction of the hydroxyl groups in ethyl gallate’s A-ring would undergo ionization (deprotonation), i.e., removal of a proton. Results from our recent study [29] suggest that the binding interaction of quercetin (a reducing cosubstrate) under partial ionization is dramatically enhanced in comparison with its non-ionizing state. Based on this interesting earlier finding, we also perform docking analysis using the partially-ionizing ethyl gallate. Predicted by *Discovery Studio*, C-3-OH of ethyl gallate has a slightly higher tendency to deprotonate than C-4-OH under physiological conditions. In the present study, we chose to determine the docking conformation when deprotonation occurs with only one hydroxyl group at any given moment, because simultaneous deprotonation of multiple protons in the same molecule are considered a rare occurrence under physiological pH conditions. 

We find that when each of the phenolic hydroxyl groups is individually deprotonated, the dominant poses (based on Δ*G*_binding_ values; Table 1) all have their ring structures inside (Figure 5D, 5G). Under C-3-OH (or C-5-OH) deprotonation (Figure 5H; Δ*G*_binding_ of −108.779 *k*_cal_/mol), the distance between the Fe ion and O^–^ is 2.254 Å. Under C-4-OH deprotonation (Figure 5E; Δ*G*_binding_ of -94.444 *k*_cal_/mol), the distance between Fe and O^–^ is 2.373 Å. This data suggests that both C-3-OH and C-4-OH can donate their electron to the Fe ion of P^+^Fe^IV^.

Potential hydrogen bonds between COX-1 and ethyl gallate in two ionizing states, as suggested by the Receptor–Ligand Hydrogen Bonds, are shown in Figure 5F and 5I. Under C-3-OH deprotonation, ethyl gallate in its dominant binding pose may form one hydrogen bond with His207 (2.420 Å); under C-4-OH deprotonation, it may form three hydrogen bonds: two with His207 (1.284 Å and 2.378 Å) and one with Gln203 (2.093 Å).

#### 3.2.2. COX-2

**Figure 6:**
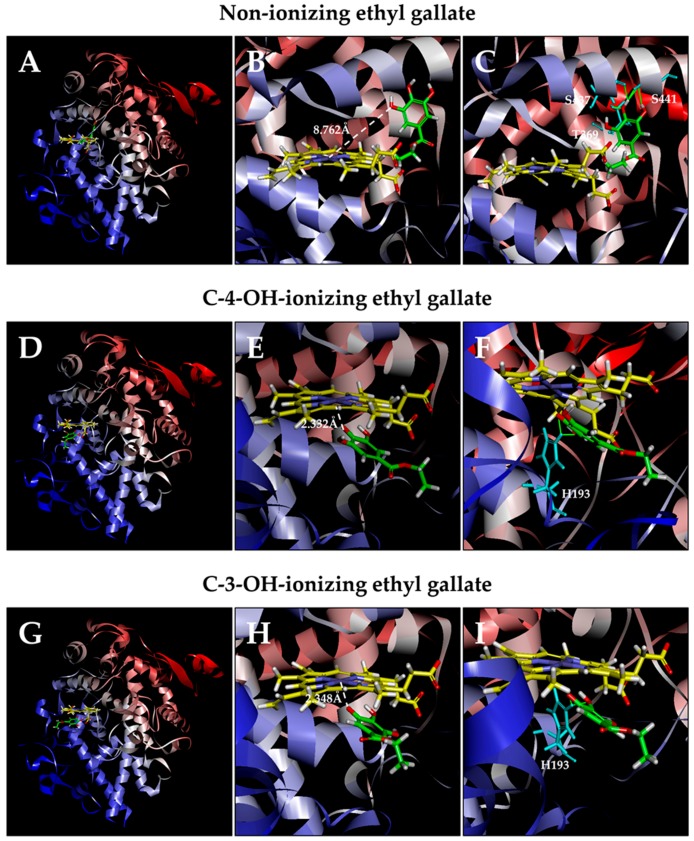
Molecular docking analysis of the binding interaction of ethyl gallate with the peroxidase active site of the COX-2 protein. Panels **A**, **D**, and **G**: The dominant docking result for non-ionizing (**A**), C-4-OH-ionizing (**D**), and C-3-OH-ionizing (**G**) ethyl gallate inside the peroxidase active site of COX-2. The protein structure is shown in a flat ribbon format. In P^+^Fe^IV^, carbon is colored in yellow, nitrogen in blue, oxygen in red, hydrogen in white, and iron in bice. In ethyl gallate, carbon is colored in green, oxygen in red, and hydrogen in white. Panels **B**, **E**, and **H**: The same structures as in panels **A**, **D**, and **G** with a white dashed line added to indicate the distance between Fe^4+^ ion and O of one of OH groups in ethyl gallate. Panels **C**, **F**, and **I**: Suggested potential hydrogen bonds (green lines) between the non-ionizing (**C**), C-4-OH-ionizing (**F**), or C-3-OH-ionizing (**I**) ethyl gallate and the amino acid residues (His193, Thr369, Ser437, Ser441) in the peroxidase site. The amino acid residues are colored in light blue.

Similar computational docking analysis was also performed with the COX-2 protein. Based on the calculated binding energy Δ*G*_binding_ values, it was predicted that the dominant binding pose of ethyl gallate in a non-ionizing state has its side chain structure inside the peroxidase site of COX-2 (Δ*G*_binding_ of −6.398 *k*_cal_/mol; Figure 6A). All hydroxyl groups in this pose are not within the catalytically-effective distance (the shortest distance from the Fe ion of P^+^Fe^IV^ is 8.762 Å; Figure 6B). For poses ranked 2–10, the hydroxyl groups of ethyl gallate are slightly farther away from the Fe ion of P^+^Fe^IV^ compared with the dominant binding pose.

We also analyzed the docking conformations when deprotonation occurs with ethyl gallate’s phenolic hydroxyl groups (Figure 6D,E). We found that when C-4-OH is deprotonated, the dominant pose has its ring structure closer to the Fe ion of P^+^Fe^IV^ (Δ*G*_binding_ of −100.929 *k*_cal_/mol; Figure 6G). In addition, the Fe ion of P^+^Fe^IV^ is very close to the O^–^ ion in ethyl gallate’s C-4-OH (2.332 Å). Under C-3-OH deprotonation, the dominant pose (Figure 6H) also has its ring inside (Δ*G*_binding_ of −132.742 *k*_cal_/mol), with a distance of 2.348 Å between the Fe ion and O^–^ ion. The suggested potential hydrogen bonds in the dominant poses in three different ionization states are shown in Figure 6C,F,I.

## 4. Discussion

In this study, we demonstrate that ethyl gallate can activate PGE_2_ production both in vitro and in vivo. It is suggested that ethyl gallate exerts this effect through the activation of the peroxidase active site of COX-1 and COX-2 by serving as a reducing cosubstrate for P^+^Fe^IV^, in a similar manner as quercetin (reported in [21,22,23,24]). This suggestion is based on the following observations: (i) The activating effect of ethyl gallate on PGE_2_ production in vitro and in vivo reported in this study closely mirrors the activating effect of quercetin reported earlier [21,23]. (ii) Activation of PGE_2_ production by ethyl gallate can be inhibited in a concentration-dependent manner by galangin, and a mirroring inhibitory effect of galangin was also observed recently with quercetin [29]. (iii) Computational docking analysis indicates that ethyl gallate can bind inside the peroxidase active sites of COX-1 and COX-2 in similar manners as quercetin, which presumably would result in re-activation of the peroxidase active sites. Moreover, our recent computational docking analysis [29] reveals that galangin can also bind inside the same peroxidase active sites of COX-1 and COX-2 in an inhibitor mode [29]. Together, these lines of data provide support for the suggestion that ethyl gallate shares a similar mechanism of action on COX-1 and COX-2 as quercetin.

Computational analysis provides useful insights into the possible mechanism by which ethyl gallate activates COX-1 and COX-2 at the molecular level. In the case of COX-1, comparison of ethyl gallate in both non-ionizing and partially-ionizing states indicates that ionization of C-3-OH or C-4-OH markedly shortens the distance between Fe^4+^ and the respective O^–^ (from 5.129 Å to 2.254 or 2.373 Å, respectively) and increases the binding affinity (Δ*G*_binding_ value decreases from −3.652 *k*_cal_/mol to −108.779 or −94.444 *k*_cal_/mol, respectively). Similarly, in the case of COX-2, ethyl gallate in the C-3-OH or C-4-OH partially-ionizing state also markedly shortens the distance between Fe^4+^ and the respective O^–^ (from 8.762 Å to 2.348 or 2.332 Å, respectively) and increases the binding affinity (Δ*G*_binding_ value decreases from −6.398 *k*_cal_/mol to −132.742 or −100.929 *k*_cal_/mol, respectively).

While C-3-OH and C-4-OH of ethyl gallate can both contribute an electron for the reduction of the COX-1 and COX-2 enzymes, differences are also noted in these two isozymes. In the case of COX-1, C-3-OH deprotonation causes the chemical to move closer to the Fe ion of P^+^Fe^IV^ than C-4-OH deprotonation. However, in the case of COX-2, C-4-OH deprotonation causes the chemical to move closer to the Fe ion of P^+^Fe^IV^ than C-3-OH deprotonation.

It is worth noting that our earlier studies [21,22,23] showed that quercetin, myricetin, and other reducing cosubstrates of the COX enzymes share a dual stimulation–inhibition phenomenon. At low concentrations, these compounds stimulate the COX enzymes as reducing cosubstrates, but at very high concentrations, they start to inhibit the catalytic activity of the COX enzymes. As explained in our earlier study [22], the main reason is because PGG_2_ (the substrate) and the reducing cosubstrate occupy the same peroxidase site. The peroxidase site needs to have the reducing cosubstrate for its re-activation after each catalytic cycle, but when the concentration of a reducing cosubstrate is too high, it would limit the access of PGG_2_ to the same site, thereby posing a net inhibitory effect on the overall catalytic activity and PG formation. Similarly, results from our present study show that ethyl gallate, 5,4′-dihydroxyflavone, and 7,4′-dihydroxyflavone also have a dual stimulation–inhibition effect. At low concentrations, they stimulate PGE_2_ production, but their effect is diminished at high concentrations or even becomes inhibitory. 

In traditional Chinese medicine, Longan is known as a fruit with “hot” properties, i.e., it has the tendency to promote inflammatory and certain other responses. However, the mechanism of its proinflammatory as well as health beneficial effects is poorly understood. The results of our present study show that ethyl gallate, a natural phenolic compound richly contained in Longan, can stimulate PG production, likely through the activation of the catalytic activity of the COX-1 and COX-2 enzymes by serving as a reducing cosubstrate for these enzymes. Similarly, a recent study from our laboratory [24] reported that ellagic acid, another phenolic compound contained in Longan and its related fruit Litchi [14,15,16,30,31,32,33], also shares this pharmacological property. It is plausible that the unique stimulatory effect of ethyl gallate and ellagic acid on PG production may partly contribute to the proinflammatory as well as health beneficial effects of Longan and Litchi.

In summary, the results of our present study show that ethyl gallate is an activator of PGE_2_ production in vitro and in vivo. Mechanistically, it is suggested that ethyl gallate exerts this effect through the activation of the peroxidase active site of COX-1 and COX-2 by serving as a reducing cosubstrate for the Fe ion of P^+^Fe^IV^. The effect of ethyl gallate is similar to the effect of some other naturally-occurring phenolics that were reported earlier [21,22,23,24]. 

## Figures and Tables

**Table 1:** Computed binding energy values (Δ*G*_binding_, *k*_cal_/mol) for the molecular docking analysis of the best binding poses between ethyl gallate (partially-ionized vs non-ionized) and cyclooxygenase 1 and 2 (COX-1 and COX-2) proteins.

Type of Protein	Binding Energy Value Δ*G*_binding_ (*k*_cal_/mol)		
	No ionization	C-4-OH ionization	C-3-OH ionization
COX-1 protein	−3.652	−94.444	−108.779
COX-2 protein	−6.398	−100.929	−132.742
